# The Versatile Roles of nc886, a Fascinating and Peculiar Regulatory Non-Coding RNA, in Cancer

**DOI:** 10.3390/ijms251910825

**Published:** 2024-10-09

**Authors:** Jiyoung Joan Jang, Dongmin Kang, Yeon-Su Lee, Yong Sun Lee

**Affiliations:** 1 Department of Cancer Biomedical Science, Graduate School of Cancer Science and Policy, National Cancer Center, Goyang 10408, Republic of Korea; wkdwldud989@gmail.com; 2 Fluorescence Core Imaging Center, Department of Life Science, Ewha Womans University, Seoul 03760, Republic of Korea; dkang@ewha.ac.kr; 3 Division of Rare Cancer, Research Institute, National Cancer Center, Goyang 10408, Republic of Korea; yslee2@ncc.re.kr

**Keywords:** nc886, oncogene, tumor suppressor, apoptosis, cancer therapy

## Abstract

This review concerns nc886, a 101-nucleotide non-coding RNA (ncRNA). Because nc886 is transcribed by RNA polymerase III (Pol III) and contains a CpG island in its promoter region, its expression is regulated by several transcription factors and the DNA methylation status. These features drive nc886 expression in two opposing directions during tumorigenesis. The known function of nc886 is to bind to and modulate the activity of target proteins such as PKR, Dicer, and OAS1. By being differentially expressed during tumorigenesis and interacting with these proteins, nc886 plays a role in tumor surveillance, promotes or suppresses tumorigenesis, and influences the efficacy of cancer therapy. The multiple roles of nc886 have been well-documented in the literature. In this review, we have summarized this literature and critically discussed the roles and mechanisms of action of nc886 in various cancers.

## 1. Introduction

### 1.1. Gene Expression, Non-Coding RNAs (ncRNAs), and Cancer

Non-coding RNAs (ncRNAs) are RNA molecules that lack an open reading frame (ORF). Although they are not translated into proteins, ncRNAs have many functions in their RNA form. For example, ribosomal RNAs (rRNAs) are structural components of ribosomes, transfer RNAs (tRNAs) decode coding information during translation, and the ncRNA (RPPH1) in the RNaseP complex plays a catalytic role in the maturation of tRNAs. In addition to these classical roles of ncRNAs in fundamental biological processes, the gene-regulatory functions of ncRNAs have been intensively studied over the last two decades. Representative classes of such regulatory ncRNAs are microRNAs (miRNAs) and long ncRNAs (lncRNAs), which control the expression of target genes mainly through base-pairing interactions with them.

When normal cells transform into abnormally proliferating, migrating and metastasizing cancer cells, genetic/epigenetic changes should occur to alter the expression and/ or activity of oncogenes and tumor suppressor genes [1]. Therefore, researchers can anticipate the contribution of these gene-regulatory ncRNAs during tumorigenesis. Driven by the technical development to detect miRNAs and lncRNAs, a large body of literature has reported their variable expression and role in many cancers [2].

Despite this boom in ncRNA research, the aforementioned classical ncRNAs, including rRNAs, tRNAs, and RPPH1, are relatively understudied. These classical ncRNAs are transcribed by RNA polymerase I or III (Pol I or Pol III), whereas miRNAs and lncRNAs are transcribed by RNA polymerase II (Pol II), the same enzyme that transcribes protein-coding genes. Because they play fundamental cellular roles, their variable expression is hardly imaginable. Several reports have documented increased activity of Pol I and Pol III in cancer cells [3], which have high metabolic demands to support their rapid, uncontrolled proliferation. Accordingly, several papers have reported the increased expression and role of ncRNAs transcribed by Pol III (hereafter referred to as “Pol III-ncRNAs”) [4]. However, the amount of literature on Pol III (or Pol I)-ncRNAs is far less than that on miRNAs and lncRNAs.

**Figure 1:**
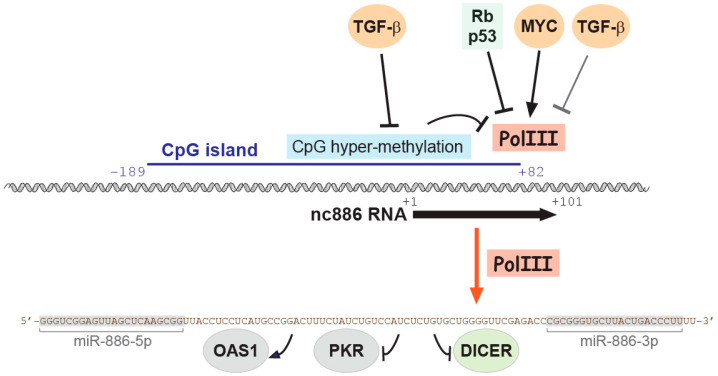
Introduction of nc886: regulatory factors for its expression and its target proteins. For each factor, its properties are indicated by color: oncogenic factors in red, tumor suppressive factors in green or blue, and neutral or ambivalent factors in grey. Arrows indicate activation and blunt arrows indicate suppression. Arrows and blunt arrows in black indicate that the relationship has been experimentally demonstrated, whereas grey color indicates no concrete evidence yet. This figure has been modified from our previous review paper [5] and edited for context.

Among Pol III-ncRNAs, we will introduce nc886 in this review, because it is a very intriguing case in terms of regulation and role (Figure 1). The regulation of nc886 expression has been extensively discussed in our recent review [5]. Here, we provide a comprehensive and critical review of the literature on the role of nc886 in cancer.

### 1.2. Introduction of nc886

The official name of nc886 is VTRNA2-1 (vault RNA2-1). In addition, nc886 was once classified as a precursor for miR-886-5p and -3p. However, experimental data to date indicate that nc886 does not produce functional mature miRNAs nor does it play a role as a component of a vault complex, a nucleoprotein complex whose function remains elusive. The identity of nc886 has been extensively discussed in our previous review [6].

So far, the known role of nc886 is to bind to target proteins and control their activity (Figure 1). These target proteins include protein kinase R (PKR, whose official name is EIF2AK2), Dicer (whose official name is DICER1), and 2′-5′-oligoadenylate synthetase1 (OAS1) (Figure 1), all of which are implicated in cancer [7,8,9]. By controlling their activities, nc886 regulates gene expression. For each of these proteins, the importance of their interaction with nc886 in the context of cancer will be discussed later. This mechanism differs from that of miRNAs and lncRNAs, which recognize target nucleic acids by base pairing [10,11]. Therefore, in the case of miRNAs and lncRNAs, it is relatively easy and straightforward to predict their target genes in silico. However, the identification of nc886’s target genes relies on bench-top experiments.

**Figure 2:**
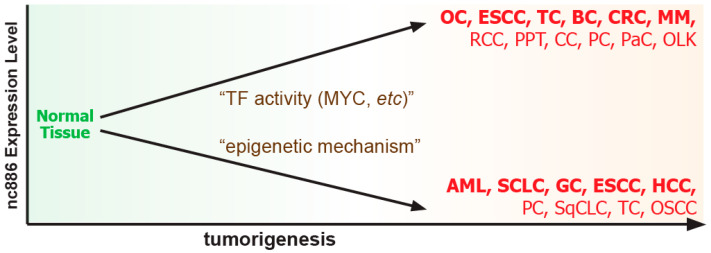
Two opposite directions of nc886 expression and their clinical significance in several cancers. Cancer types in plain letters represent those in which nc886 expression has been documented in clinical samples. Cancer types in bold are those for which an association of nc886 expression with patient prognosis has been documented [5]. Abbreviations for each cancer type are as follows: ovarian cancer, OC; esophageal squamous cell carcinoma, ESCC; thyroid cancer, TC; bladder cancer, BC; colorectal cancer, CRC; multiple myeloma, MM; renal cell carcinoma, RCC; prolactin pituitary tumor, PPT; cervical cancer, CC; prostate cancer, PC; pancreatic cancer, PaC; oral leukoplakia, OLK; acute myeloid leukemia, AML; small cell lung cancer, SCLC; gastric cancer, GC; hepatocellular carcinoma, HCC; squamous cell lung carcinoma, SqCLC; oral squamous cell carcinoma, OSCC. This figure has been modified from our previous review paper [5] and edited for context.

Due to its transcription by Pol III, nc886 is abundantly expressed in most tissues [5,12], suggesting that nc886 is involved in most cancer types, regardless of the tissue or organ from which they arise. The expression of nc886 is increased or decreased during tumorigenesis through regulation by oncogenic or tumor suppressive transcription factors (TFs) and epigenetic mechanisms (Figure 1 and Figure 2) (reviewed in [5]). These two opposite directions of nc886 expression in cancer suggest that nc886 may have an oncogenic or tumor-suppressing effect, depending on cancer types and individual tumors.

## 2. The Role of nc886 in Cancer

The expression patterns of nc886 and its target proteins strongly suggest multiple roles for nc886 in cancer (Figure 1 and Figure 2). In this review, we will summarize all papers that have investigated nc886 in cancer using gain-of-function and loss-of-function approaches and critically evaluate their conclusions. When discussing individual papers, we will also describe the experimental methods they use for proper evaluation. Although the issue of correct gain-of-function and loss-of-function methods has been described in detail in our previous review [6], we will briefly describe again below.

A legitimate way to overexpress nc886 is to clone the nc886 RNA and its flanking genomic sequence into a plasmid lacking a mammalian promoter, so that nc886 is expressed from its own promoter. An equally legitimate way is to insert the nc886 RNA sequence under an external Pol III promoter such as U6 and H1 [13,14,15]. An alternative method is to transfect in vitro synthesized nc886 RNA molecules [16,17].

For nc886 knockdown (KD), the most reliable method to date is the use of a backbone-modified antisense oligonucleotides (anti-oligo) [18,19,20]. The efficient KD by this modified anti-oligo has been undoubtedly demonstrated by northern blot, in which the disappearance of the nc886 band was observed after transfection of it [12,21]. In one study [22], a small interfering RNA (siRNA) was used for nc886 KD. However, they did not confirm KD efficiency by measuring nc886 levels, nor did they observe any effect on cells.

It is of note that a significant proportion of the papers aimed to study miR-886-5p or -3p. We have carefully evaluated their experimental approaches and included them in this review if they unintentionally overexpress or repress nc886. For example, an anti-oligo targeting miR-886-5p (or -3p) [23,24,25] could bind to nc886 and be considered a loss-of-function experiment of nc886. Some studies transfect plasmid containing the nc886 sequence as a gain-of-function approach for miR-886-5p (or -3p) [23,26], assuming that nc886 would produce miR-886-5p (or -3p).

### 2.1. nc886 as a Tumor Surveillant

**Figure 3:**
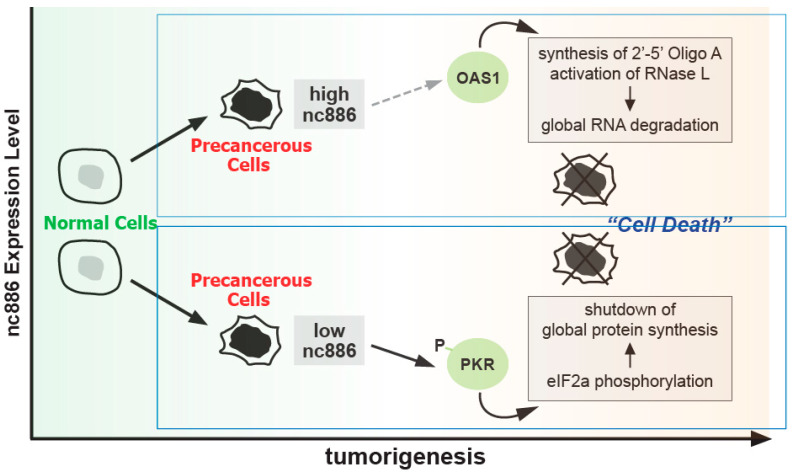
The role of nc886 in tumor surveillance. In the OAS1 part (top, thin blue box), the dotted grey arrow indicates an uncertain link that requires further evidence. The PKR part is in a thicker box, indicating that it has more experimental evidence. 2′-5′ Oligo A, 2′-5′-linked oligoadenylate; eIF2α, the α subunit of the eukaryotic initiation factor 2. See text for details.

This role of nc886 is based on its interaction with PKR. A well-established function of PKR is the induction of apoptosis when activated by sensing intrusion of pathogens. The molecular events underlying this phenomenon are as follows: Pathogen-driven double-stranded RNA (dsRNA) binds to PKR with high affinity and activates PKR. The active PKR phosphorylates the α subunit of the eukaryotic initiation factor 2 (eIF2α, whose official name is EIF2S1), shuts down global translation, and ultimately induces apoptosis (reviewed in [27]) (Figure 3). This cell death, the elimination of pathogens together with infected cells, is an important event for the health of a human organism.

It is known that nc886 binds to PKR with high affinity (K_D_ = 12.3 nM), which is comparable to PKR’s canonical activator, dsRNA (K_D_ = 4.0 nM) [28]. In contrast to dsRNA, PKR is kept inactive when bound to nc886. Based on the K_D_ value and the copy numbers of PKR and nc886, most PKR molecules in normal cells are estimated to be an nc886-bound, inactive form. This is well evidenced by PKR activation and resultant cell death upon nc886 KD in various cell lines (Table 1).

The nc886 gene is expressed in most normal human tissues and epigenetic silencing occurs in a significant proportion of cancer cells (to be elaborated below). Taking these facts together with the nc886-PKR cell death pathway, we have proposed a tumor surveillance model (reviewed in [35]). In this model, CpG hypermethylation at the nc886 promoter and consequent silencing of nc886 expression in a cell is sensed as a pre-cancerous event, and the resultant PKR activation leads to apoptosis to eliminate such a cell (Figure 3). This parallels the role of PKR in innate immunity, where dsRNA is perceived as pathogen evasion and the resulting activation of PKR induces apoptosis to eliminate an infected cell.

The silencing of nc886 expression is the initial, triggering event in the nc886-PKR tumor surveillance model. The efficacy of the surveillance could be questioned, because nc886 silencing occurs through CpG hypermethylation at the nc886 promoter, which is thought to be an uncommon event. Furthermore, as most normal cells have two alleles, aberrant hypermethylation must theoretically occur in both alleles for complete silencing of nc886 and consequent activation of PKR. However, this is partially true for a minor fraction of the human population, due to unique features of nc886 epigenetics. Importantly, nc886 is a maternally imprinted gene. This imprinting is polymorphic and occurs in approximately 75% of human individuals. In other words, in these 75%, the nc886 promoter is CpG hypermethylated in the maternal allele and so nc886 is transcribed only from the paternal allele. In the remaining 25% of individuals, both alleles are hypomethylated and can contribute to nc886 expression. This is important in predicting the likelihood of nc886 silencing during tumorigenesis. In the 75% of individuals, aberrant CpG hypermethylation on the paternal allele alone is sufficient for complete silencing of nc886 expression. Therefore, nc886 silencing during tumorigenesis is expected to be far more common than most other autosomal genes. Considering this feature of nc886, we speculate that the nc886-PKR tumor surveillance eliminates a significant number of pre-cancerous cells, although it is almost impossible to estimate the effectiveness of the surveillance because those eliminated cells would not be detectable.

So far, we have discussed nc886′s tumor surveillance role based on its regulation of PKR, an anti-viral and pro-apoptotic protein. A similar case has been reported in one study, namely OAS1 [17]. Like PKR, OAS1 is a pathogen sensor that recognizes dsRNA. Once activated by dsRNA, OAS1 synthesizes the 2′-5′-linked oligoadenylate (“2′-5′ Oligo A” in Figure 3) second messenger and thereby activating RNase L, which degrades viral and cellular RNAs (reviewed in [36], Figure 3). These events lead to apoptosis. According to the work from the Conn laboratory [17], an nc886 mimic RNA, which is synthesized by in vitro transcription, activates OAS1 both when mixed with purified OAS1 in vitro and when transfected into A549 cells. Although apoptosis was not measured in this study, several other studies have observed that transient transfection of an nc886 mimic RNA induces apoptosis or inhibits cell proliferation in esophageal squamous cell carcinoma (ESCC) cells, gastric cancer (GC) cells, thyroid cells, and colon cancer cells [16,32,37]. This cytotoxic or anti-proliferative effect might be due to the activation of OAS1. Together with the increased expression of nc886 in cancer, these data suggest another tumor surveillance model in which the increased nc886 during tumorigenesis activates OAS1 and induces apoptosis to eliminate pre-cancerous cells (Figure 3). However, it is questionable whether this mechanism actually operates during tumorigenesis. Firstly, the dose of the nc886 mimic used in the study [17] to activate OAS1 was about 10^8^ copies per cell, which is much higher than the intracellular concentration of nc886, which ranges from 10^4^ to 10^5^. Secondly, the increase in nc886 would occur gradually over a long period in tumorigenesis, which is clearly different from the sudden administration of nc886 to cells in transfection experiments. Nevertheless, once proven, the nc886-OAS1 tumor surveillance mechanism would occur in many cells, as nc886 expression is generally increased in cancer.

### 2.2. nc886′s Oncogenic Roles

As mentioned above, nc886 expression is increased in cancer due to a general increase in Pol III activity (Figure 1 and Figure 2), suggesting an oncogenic role. This notion was supported by clinical data in various cancer types. In these cancers, patients with high expression of nc886 have more advanced cancer stage, higher Gleason score, and worse prognosis, including shorter survival (reviewed in [5]). These data are not direct evidence but a clinically important finding.

The strictest definition of an oncogene is the ability to drive tumorigenesis when overexpressed or hyper-activated. An ideal experiment will be to make a transgenic mouse that overexpresses nc886 and to observe whether the mouse is predisposed to cancer. However, there is no nc886 orthologue in mice [38,39]. It is therefore questionable whether nc886 could have an orthologous function in tumorigenesis when xenogeneically expressed in mice.

A more practical approach is to use patient-derived xenograft (PDX) or cell line-derived xenograft (CDX) models. However, there is also a hurdle. Based on experiments in human mammary epithelial cell (HMEC) lines, which are an in vitro system that mimics breast tumorigenesis, nc886 expression is increased approximately 10-fold in malignant cells compared to normal cells [40]. This increase is due to MYC proto-oncogene, bHLH transcription factor (MYC), a TF whose oncogenic role is well established [41]. For correct experimental design, nc886-overexpressing normal cells must be generated and these cells must be isogenic to the parental normal cells, with the only difference being nc886. To achieve this, the sequence of the nc886 DNA region should be manipulated so that nc886 is expressed from a stronger promoter. However, such manipulation is challenging, because nc886 is a Pol III-ncRNA. The gene-internal promoter elements of nc886 create an inherent limitation to changing the promoter sequence. Replacement with a promoter of another Pol III-ncRNA could not be a solution, because the nc886 promoter is not inferior, as judged by the sequences of its promoter elements which are not deviant from the consensus sequence [5]. It should be recalled that the high-level expression of nc886 in cancer cells is due to MYC and the resultantly elevated activity of the Pol III enzyme, which is expected to increase Pol III-ncRNAs globally [42]. In conclusion, to the current knowledge, it is almost impractical to generate a cell line from normal cells that overexpresses only nc886.

**Figure 4:**
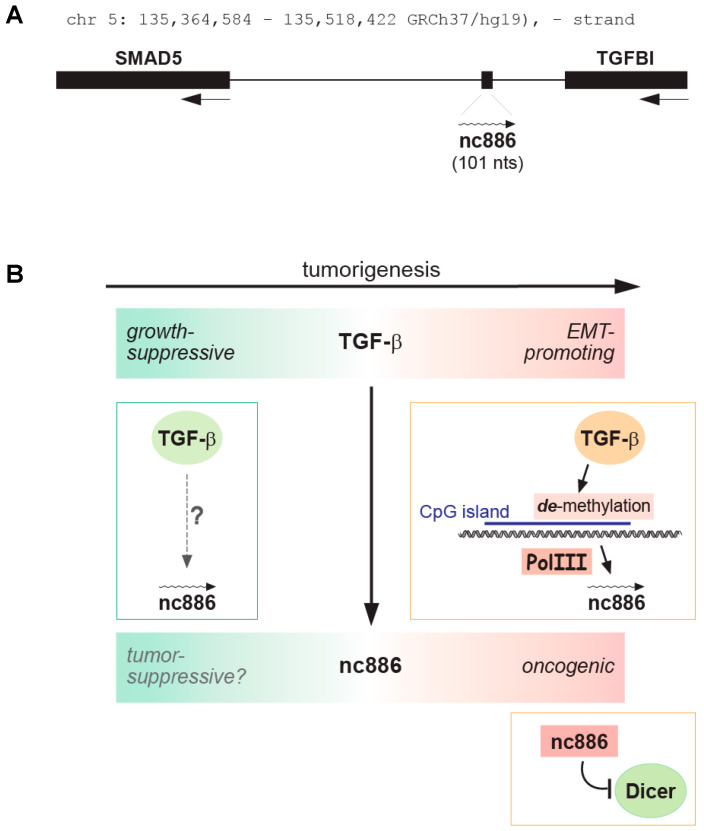
The roles of nc886 and TGF-β during tumorigenesis. (**A**). A cartoon depicting the genomic locus for nc886, TGFBI, and SMAD5. Arrows indicate the direction of transcription. Except for nc886, the size of the genes and their intervals are drawn to scale. (**B**). Oncogenic properties are colored in red and tumor suppressive properties are in green. An arrow indicates activation and a blunt arrow indicates inhibition. A dotted grey arrow indicates an uncertain event that needs more experimental evidence. See text for details.

Nevertheless, several reports have shown that nc886 contributes to tumorigenesis. The most prominent is its role in the transforming growth factor-β (TGF-β) signaling pathway. The relationship between nc886 and TGF-β was inferred by the genomic locus of nc886, which is flanked by TGFBI and SMAD5, both of which are implicated in the TGF-β pathway (Figure 4A). TGF-β is an important player in cancer and is known to play two opposing roles during tumorigenesis [43]. TGF-β acts as a tumor suppressor in early stages, but promotes cell invasiveness and motility in later stages (Figure 4B). A study in ovarian cancer (OC) [14] has shown that nc886 expression is induced by TGF-β via an epigenetic mechanism. Upon induction, nc886 suppresses the miRNA pathway by inhibiting Dicer, to enhance adhesion, migration, and invasion of OC cells (Figure 4B and Table 2). In addition, high expression of nc886 correlates with shorter survival of OC patients. All these data provide compelling evidence for an oncogenic role of nc886 in OC, especially at later stages (Figure 4B). It should be noted that the cell culture data in this study were obtained by ectopic expression of nc886 in nc886-silenced OC cells.

Taken together, this study and our knowledge of nc886 suggest that the expression and role of nc886 during OC tumorigenesis is as follows (Figure 4B). Normal ovarian surface epithelial (OSE) cells express nc886. When nc886 is silenced, most pre-cancerous OSE cells would have died through the nc886-PKR tumor surveillance mechanism, but a certain fraction of OSE cells survive and develop into OC cells. At this early stage, nc886 is possibly a tumor suppressor and its silencing could promote OC development. Then, at some point, TGF-β reverses nc886 silencing by CpG demethylation, thereby facilitating progression to more aggressive OC. According to this scenario, the role of nc886 coincides with that of TGF-β, which is a tumor suppressor in early stages but becomes an oncogene in later stages. The data that TGF-β demethylated nc886 in OC cells raises an interesting question of whether nc886 silencing in normal OSE cells is caused by low TGF-β activity or is simply a stochastic event.

Some studies have argued for an oncogenic role of nc886 based on the observation that loss of function of nc886 leads to decreased proliferation or induction of apoptosis [29,30]. Conceivably, this observation could be evidence for a pro-proliferative or anti-apoptotic role, which is a feature of oncogenes. However, in the case of nc886, the loss-of-function data should be interpreted in the context of the tumor surveillance model (see Table 1). In this model, nc886 KD activates PKR and induces apoptosis, as observed in the above studies. In this context, the KD data do not guarantee that cell proliferation or apoptosis is affected by the increase in nc886 expression, which occurs during tumorigenesis. PKR is already kept inactive in most normal cells and therefore will not be further suppressed by an increase in nc886 expression. In light of this, it is unlikely that PKR is involved in a cellular phenotype induced by the gain-of-function of nc886.

Although a loss-of-function approach has the caveat of PKR-mediated cell death, several studies have circumvented this and successfully demonstrated an oncogenic role for nc886 (Table 2). One study was in thyroid cancer (TC) [31] and the other in oropharyngeal squamous cell carcinoma (OPSCC) [44]. In these two studies, the initial intention was to perform an nc886 knockout (KO) using a technique called “clustered regularly interspaced short palindromic repeats (CRISPR) and CRISPR-associated protein (Cas9)”. However, nc886 KO cells could not be obtained in the presence of PKR but were produced in a PKR KO background. These failures and successes can be well explained by the nc886-PKR cell death pathway.

**Table 2:** Cell culture data showing the oncogenic role of nc886.

Cancer Type	Approach	Method	Cellular Phenotype upon Ectopic Expression or KD of nc886	Ref.
ovarian cancer (OC)	ectopic expression	plasmid-based (from Pol III promoters)	- increase in attachment, migration and invasion of SKOV3 and A2780 cells	[14]
	KD	modified anti-oligo *	- decrease in TGF-b-induced migration of OSE80PC cells	
renal cell carcinoma (RCC)	ectopic expression	synthetic nc886 mimic RNA **	- increase in cell viability and invasion of A-498 cells	[45]
			- decrease in apoptosis of A-498 cells	
	KD	synthetic nc886 inhibitor RNA **	- decrease in cell viability and invasion of A-498 cells	
			- increase in apoptosis of A-498 cells	
thyroid cancer (TC)	KO	CRISPR-Cas9 ***	- decrease in proliferation, migration and invasion and colony formation of nc886 KO Nthy-ori 3-1 cells ***	[31]
oropharyngeal squamous cell carcinoma (OPSCC)	KO	CRISPR-Cas9 ***	- decrease in proliferation of FaDu cells ***	[44]

* The modified anti-oligo as described in Table 1. ** these mimic and inhibitor are assumed to be synthetic RNAs, but their molecular identities are not specified in the reference. *** nc886 KO in the PKR KO background, and so the comparison of phenotype is between nc886/PKR double KO cells vs. the parental PKR KO cells.

In TC, the KO experiment was performed in Nthy-ori 3-1, an immortalized but not yet malignant cell line [46]. The nc886-PKR double KO cells were less proliferative, migratory, and invasive than the parental PKR KO cells [31] (Table 2). Thus, these data represent a tumor-promoting role of nc886 during immortalization, which is a relatively early stage of tumorigenesis. In OPSCC, the nc886-PKR double KO FaDu cells proliferated slowly as compared to the parental PKR KO cells [44] (Table 2). FaDu is a malignant cell line, based on its origin and ability to form tumors in xenografts [47,48]. Given that nc886 expression is generally increased in cancer, these data suggest that increased nc886 expression is one way of supporting the rapid proliferation of cancer cells.

When complemented with the loss-of-function experiments, gain-of-function experiments provide more credible data. One example is a study in renal cell carcinoma (RCC) [45] (Table 2). Upon transfecting an nc886 mimic, which is presumed to be a synthetic RNA, into an RCC cell line, they observed an increase in cell viability and invasion and a decrease in apoptosis. These mimic data were complemented by experiments with an nc886 inhibitor, which showed exactly the opposite results to the mimic. Upon transfection of the mimic and the inhibitor, they also measured the phosphorylation of JAK2 and STAT3, which are known to be oncogenic [49], to claim they are targets of nc886 for its oncogenic role in RCC. However, the fold-change in JAK2 and STAT3 phosphorylation was very modest. It should be noted that caution should be exercised as to whether the nc886 mimic and inhibitor legitimately represent the gain- or loss-of function of nc886, as there is no detailed information on their molecular identity.

### 2.3. nc886′s Tumor Suppressive Roles

As previously stated, nc886 is expressed in most normal cells and has CpG islands in the promoter region. When aberrantly hypermethylated, nc886 expression is silenced. These phenomena are also seen in many tumor suppressor genes [50,51], suggesting that nc886 is also a tumor suppressor. Indeed, CpG hypermethylation in the nc886 promoter region and consequently decreased nc886 RNA levels have been observed in a number of cancer types including GC, ESCC, prostate cancer (PC), small cell lung cancer (SCLC), and acute myeloid leukemia (AML) [26,32,37,52,53]. Notably, the increased methylation and/or decreased expression of nc886 is quite common in patients, despite the globally increased expression of Pol III-ncRNAs due to elevated Pol III activity in cancer. Data from patients show worse prognosis such as more advanced tumor stage, shorter survival, when nc886 methylation is high or nc886 RNA levels are low, providing clinical evidence that nc886 is a tumor suppressor (reviewed in [5]).

Not only decreased in cancer patients, nc886 expression is silenced in a significant fraction of cancer cell lines (approximately 30% based on unpublished data from our laboratory). This frequency is astonishingly high when one considers the conditions under which nc886-silenced cells arise. First, aberrant CpG hypermethylation must occur. Second, nc886-silenced cells must survive the tumor surveillance mechanism. As discussed earlier, nc886 is imprinted in 75% of the human population, so the event of nc886 silencing would be much more common than most autosomal genes. However, this feature alone is not sufficient to explain the incredibly high proportion of nc886-silenced cells. This proportion can be explained by the assumption that they have acquired pro-survival and pro-proliferative properties and have selectively outgrown.

This assumption is supported by gene expression profiling upon nc886 KD in gastric, esophageal cell lines [32,37] (Table 3). Although an obvious cellular phenotype upon nc886 KD is apoptosis, the most prominent change in gene expression of these nc886-depleted cells is the induction of well-known oncogenes, including MYC, FOS and MAFB, and the activation of the oncogenic nuclear factor kappa-light-chain-enhancer of activated B cells (NF-κB) pathway. As these pro-inflammatory oncogenes are downstream branches of the PKR pathway, their induction is presumably due to the activation of PKR by nc886 KD. These data suggest that nc886-silenced cells have a strong potential to develop into cancer cells once they bypass the PKR-eIF2α cell death, which is another consequence of nc886 depletion [35]. In addition to the KD data, ectopic expression of nc886 also suppresses the NF-κB pathway in the presence of several NF-κB-activating triggers [12,54]. In addition to transcriptome analysis, a proteomics approach identified Akt, a well-established pro-survival pathway in cancer [55,56], to be activated upon nc886 KD in an ESCC cell line [37]. Overall, nc886 plays a tumor suppressor role, presumably by repressing a variety of oncogenes, including mainly pro-inflammatory ones.

With nc886 KD in cell culture, it is difficult to obtain experimental evidence of an enhanced tumorigenic phenotype, due to acute cell death. Instead, gain-of-function approaches have provided experimental evidence (Table 3). When nc886 is stably expressed in 293T cells, these nc886-expressing 293T cells stay longer in the G1 phase of the cell cycle and resultantly proliferate more slowly than the nc886-silenced control 293T cells [13]. This slower growth is likely to be due to the altered expression of cell cycle genes, presumably caused by the suppression of Akt by nc886. A similar result was obtained in PC cell lines [52]. When nc886 is overexpressed, DU145 and LNCaP PC cells have altered expression of cell cycle genes and stay longer in the G2-M phase. As a result, these PC cells proliferate slowly when cultured in vitro and when injected into nude mice.

In PC, nc886 also suppresses tumor invasiveness, as indicated by reduced invasion of the nc886-expressing DU145, LNCaP, and PC-3M-1E8 cells [15,52]. This finding is in line with clinical data showing higher nc886 methylation and lower nc886 expression in metastatic tumors than in normal tissues or primary tumors. In a more detailed study [15], nc886-expressing PC-3M-1E8 cells have shown more epithelial characteristics than the original mesenchymal PC-3M-1E8 cells. TGF-β, an inducer of epithelial-to-mesenchymal transition (EMT), restores the mesenchymal character, when treated in nc886-expressing PC-3M-1E8 cells. The TGF-β treatment also resulted in low expression of nc886. This reduced expression is probably due to a general decrease in Pol III enzyme activity, because nc886 expression in PC-3M-1E8 cells was driven by a heterologous H1 promoter. Regarding the regulation of nc886 in PC, OC and other cancers, the role of TGF-β in CpG methylation and the Pol III enzyme activity needs to be elucidated.

### 2.4. The Effect of nc886 in the Cancer Therapy

Several therapeutic modules, including chemotherapy, immunotherapy, and gene-targeting therapy, are used to selectively kill cancer cells. As discussed in previous sections, nc886 controls innate immunity and determines cell death. It is therefore not surprising that nc886 is a key determinant of a cell’s response to cancer therapies. This notion is supported by clinical data comparing the response to chemotherapy after grouping patients according to nc886 expression levels. Patients with high nc886 are more resistant to chemotherapy in OC [14]. Conversely, nc886-high patients are more sensitive to chemotherapy in colon cancer [57].

There are several cell culture experiments showing nc886′s effect on drug efficacy (Table 4). An OC cell line, SKOV3, is nc886-silenced and has an IC_50_ value of ~0.05 μM when treated with paclitaxel, a commonly used chemotherapeutic agent in OC patients [14]. In comparison, SKOV3 cells stably expressing nc886 have a significantly higher IC_50_ (~0.35 μM). In terms of paclitaxel sensitivity, nc886 was investigated in a cervical cancer (CC) cell line, SiHa [58]. In this study, they transfected with an anti-oligo against nc886 and claimed that more cells underwent apoptosis upon nc886 KD than the negative control, when treated with paclitaxel. However, it should be recalled that nc886 KD by itself induces apoptosis, as shown also in this study. Therefore, the increased sensitivity of nc886-depleted SiHa cells could simply be an additive effect of nc886 KD and paclitaxel. Another study examined the sensitivity to sunitinib and everolimus, which are drugs used to treat RCC [59]. In the presence of these drugs, they observed that RCC cell lines died more with nc886 KD and survived more with nc886 overexpression. However, this study did not provide data on the effect of nc886 KD or overexpression per se in the absence of the drugs, and so a simple additive effect therefore remains a possibility. In addition, detailed information on nc886 KD and overexpression was not provided in these CC and RCC studies, making it difficult to evaluate their data.

Chemotherapeutic drugs are mostly DNA-reactive compounds and are therefore thought to target only proliferating cells with DNA replication. However, a long-standing question is whether and how they also damage non-replicating normal cells. A study on nc886 elucidated this question [16]. This study discovered a novel role for doxorubicin, a chemotherapeutic drug. Doxorubicin evicts the Pol III enzyme from DNA almost immediately after treatment. Combined with the fact that nc886 is very unstable RNA with a half-life of approximately 1 to 2 h, doxorubicin depletes nc886, thereby activating PKR and inducing cell death. These events occur even before the DNA damage response induced by doxorubicin treatment. Importantly, since the nc886-PKR cell death pathway operates in most non-proliferating normal cells, this mechanism explains why doxorubicin is cytotoxic to them.

Tumor suppressor genes are often silenced by CpG DNA hypermethylation. In principle, DNA-demethylating agents such as 2′-deoxy-5-azacytidine (DAC) would re-activate them and are therefore used in cancer therapy. In addition to this original rationale, DAC treatment induces the transcription of repeat elements and leads to the generation of endogenous dsRNA, which activates PKR and induces apoptosis with different efficacy between cells. A recent study attempted to explain this difference with nc886 [60]. In their experiments with the MCF-7 cell line which is nc886-silenced (based on unpublished data from the YSL laboratory), DAC treatment induces nc886 expression. While DAC alone is not as cytotoxic, DAC combined with nc886 KD significantly reduces cell viability. These data suggest that the induced nc886 attenuates dsRNA-mediated cytotoxicity by competing for PKR and keeping it inactive. To elucidate nc886′s effect on DAC-mediated cytotoxicity, data should be collected from a variety of cell lines with different nc886 expression and PKR activity.

## 3. Conclusions and Future Perspectives

As we have summarized a number of literatures in this review, there is no doubt that nc886 is an important player in cancer. It is also clear that the role of nc886 in cancer is complicated and cannot be defined in one direction. The expression levels of nc886 increase or decrease during tumorigenesis. Clinical data from cancer patients show conflicting results; poor prognosis is associated with high expression of nc886 in some types of cancer, but with low expression in others. This ambivalent role of nc886 is also seen in cell culture studies reporting more tumorigenic phenotypes upon nc886 overexpression in some literatures but upon nc886 KD in others. This ambivalence is also seen in important genes/pathways in cancer, including MYC, NF-κB, TGF-β, and Notch [61,62,63,64].

At the molecular level, this ambivalence can be explained by the target proteins that nc886 controls. For example, PKR can be pro-apoptotic or pro-proliferative, depending on which downstream pathway is dominantly in effect. Dicer is required for the biogenesis of all miRNAs, and each miRNA plays a different role. In addition to PKR and Dicer, nc886 interacts with many other proteins, including OAS1 and HuR (whose official name is ELAVL1) [25]. The role of nc886 in a cell in a given situation would be determined by which protein dominantly interacts with nc886 and which downstream pathway is activated or suppressed. All these events are ultimately dictated by the gene expression profile which would differ between individual cancer cells.

It appears that the exact expression level and role of nc886 varies depending on cancer types, individual patients, cancer stages, etc. Despite this complexity, we can address several facts that are relatively certain, based on the consistency of published results on nc886 data and the general knowledge. As previously stated, nc886 is expressed in most normal human tissues. Although nc886 expression is increased during tumorigenesis, nc886 is frequently silenced due to its epigenetic characteristics. Silencing of nc886 mostly results in cell death, but also induces pro-inflammatory oncogenes. These facts underlie the nc886-PKR tumor surveillance model (see Section 2.1). Whether nc886 actually contributes to reducing cancer incidence will require experimental evidence, which is very challenging to obtain.

From a clinical perspective, nc886 appears to be unsuitable as a universal marker for cancer diagnosis due to its dual expression pattern. Furthermore, the unstable nature of nc886, with a half-life of approximately 1 to 2 h [5], is another obstacle to its use as a molecular marker. However, several lines of evidence have undoubtedly shown that the expression level of nc886 is a useful information in predicting a patient’s prognosis and response to cancer therapy. To exploit nc886 in the clinic, future studies are needed to understand how nc886 expression is regulated, what function nc886 exerts, and what cellular role it plays in a given biological context. This will require the accumulation of a large body of reliable data generated using legitimate experimental methods.

## Figures and Tables

**Table 1:** Studies showing PKR activation and subsequent decrease of cell viability upon nc886 KD.

Organ or Cell Type	Methods for nc886 KD	Results (Events of Cells upon nc886 KD)	Ref.
endometrium	siRNA	- increase in apoptosis of HEC-1A cells	[29]
renal cell	synthesized miR-886-3p inhibitor (GenePharma)	- decrease in migration and proliferation of 786-O and ACHN cells	[30]
		- increase in apoptosis of 786-O and ACHN cells	
thyroid	modified anti-oligo *	- PKR activation and decrease in cell viability of Nthy-ori 3-1 and SW1736 cells	[31]
stomach	modified anti-oligo *	- decrease in proliferation of HFE-145, SNU-601, and SNU-638 cells	[32]
		- increase in apoptosis of SNU-601 and SNU-638 cells	
		- PKR activation and the downstream cell death in HFE-145 cells	
bile duct	modified anti-oligo *	- PKR activation in M156 and M214 cells	[33]
		- PKR activation and the downstream cell death in MMNK1 cells	
various	modified anti-oligo *	- PKR activation in Nthy-ori 3-1 and HCT-116 cells	[16]
		- increase in apoptosis of Nthy-ori 3-1 and HCT-116 cells	
		- decrease in cell viability of Nthy-ori 3-1 and HCT-116	
various	modified anti-oligo *	- decrease in cell proliferation of HeLa, HCT-116, CRL2741, WI-38, and MDA-MB231 cells	[12]
		- PKR activation in HCT-116 cells	
CD4+ T cell	pre-miR-886 inhibitor (Qiagen) **	- decrease in cell proliferation of HuT-78 cells stimulated with CD3/D28 activation signals	[22]
keratinocyte	modified anti-oligo *	- PKR activation in HaCaT cells	[34]

* The modified anti-oligo whose KD efficacy is experimentally validated as described in the main text. It is a backbone-modified (to phosphorothioate) 20-mer anti-oligo containing ten central deoxyribonucleotides flanked by five 2′-O-methoxyribonucleotides at both termini. ** locked-nucleic acid.

**Table 3:** Cell culture data showing the tumor suppressor role of nc886.

Cancer Type	Approach	Method	Cellular Phenotype upon Ectopic Expression or KD of nc886	Ref.
prostate cancer (PC)	ectopic expression	plasmid-based (from the CMV promoter)	- decrease in proliferation and invasion in DU145 and LNCaP cells	[52]
			- delayed cell cycle at the G2/M phase in DU145 cells	
			- decrease in tumor growth when xenografted into BALB/c nude mice	
	ectopic expression	plasmid-based (from the H1 promoter)	- increase in colony formation of PC-3M-1E8 cells	[15]
			- decrease in migration and invasion of PC-3M-1E8 cells	
			- decrease in bone metastasis when xenografted into BALB/c nude mice	
gastric cancer (GC)	ectopic expression	plasmid-based (from Pol III promoters)	- increase in apoptosis of MKN-01 and SNU-484 cells	[32]
		synthetic nc886 RNA (in vitro transcripts)	- decrease in proliferation of MKN-01 and SNU-484 cells	
	KD	modified anti-oligo *	- induction of oncogenes (FOS, NF-kB target genes, MYC) in HFE-145 cells	
esophageal squamous cell carcinoma (ESCC)	ectopic expression	plasmid-based (from Pol III promoters)	- decrease in proliferation of 293T cells **	[13]
			- delayed cell cycle at the G1 phase in 293T cells **	
	ectopic expression	synthetic nc886 RNA (in vitro transcripts)	- decrease in proliferation of TT and TE-12 cells	[37]
			- increase in apoptosis of TT and TE-12 cells	
	KD	modified anti-oligo *	- induction of oncogenes (FOS, MYC, NF-kB target genes, MAFB, ID2) in Het-1A, TE-1, and TE-8 cells	
small cell lung cancer (SCLC)	ectopic expression	plasmid-based ***	- decrease in proliferation of NCI-H446 cells	[26]
			- decrease in tumor growth with no sign of tumor invasion and metastasis when xenografted into BALB/c nude mice	

* The modified anti-oligo as described in Table 1. ** phenotypes were observed 293T cells, which are not an ESCC cell line, as an alternative after justifying from gene expression profiles *** 500 nucleotides containing nc886 and the flanking sequences were cloned under the CMV promoter.

**Table 4:** The effect of nc886 on cancer therapeutic drugs.

Cancer Type	Therapeutic Drug	nc886 Expression upon Drug Treatment	Approach and Method	Change in Drug Sensitivity upon Ectopic Expression or KD of nc886	Ref.
cervical cancer (CC)	paclitaxel and VP16	increase	nc886 KD by anti-oligo *	- increase in paclitaxel-induced cell death of SiHa cells	[58]
not specific	2′-deoxy-5-azacytidine (DAC)	increase	nc886 KD by siRNA	- increase in DAC-induced cell death of MCF-7 cells	[60]
renal cell carcinoma (RCC)	sunitinib and everolimus	decrease	ectopic expression by transfecting a plasmid that expresses nc886	- decrease in sunitinib- and everolimus-induced cell death of 786-O and ACHN cells	[59]
				- increase in sunitinib- and everolimus-induced EMT of 786-O and ACHN cells	
			nc886 KD by siRNA	- increase in sunitinib- and everolimus-induced cell death of 786-O and ACHN cells	
				- decrease in sunitinib- and everolimus-induced EMT of 786-O and ACHN cells	
not specific	doxorubicin	decrease	ectopic expression by transfecting the in vitro transcript of nc886	- decrease in doxorubicin-induced cell death of HCT-116 and Nthy-ori 3-1 cells	[16]
esophageal squamous cell carcinoma (ESCC)	palbociclib		nc886 KD by anti-oligo *	- increase in palbociclib-induced cell death of TE-1 cells	[13]
ovarian cancer (OC)	paclitaxel		ectopic expression (by generating a cell line stably expressing nc886)	- decrease in paclitaxel-induced cell death of SKOV3 cells	[14]

* The modified anti-oligo as described in Table 1.
